# Targeting MDM2-p53 interaction for breast cancer therapy

**DOI:** 10.32604/or.2025.058956

**Published:** 2025-03-19

**Authors:** AMJAD YOUSUF, NAJEEB ULLAH KHAN

**Affiliations:** 1 Clinical Laboratory Sciences Department, College of Applied Medical Sciences, Taibah University, Madinah, 41477, Saudi Arabia; 2 Institute of Biotechnology and Genetic Engineering, The University of Agriculture, Peshawar, Peshawar, 25130, Pakistan

**Keywords:** Breast cancer, Murine double minute 2 proto-oncogene (MDM2), Tumor protein 53(TP53), Targeted therapy

## Abstract

Breast cancer is a significant global concern, with limited effective treatment options. Therefore, therapies with high efficacy and low complications, unlike the existing chemotherapies, are urgently required. To address this issue, advances have been made in therapies targeting molecular pathways related to the murine double minute 2 proto-oncogene (MDM2)-tumor proteinp53 (TP53) interaction. This review aims to investigate the efficacy of MDM2 inhibition in restoring TP53 activity in breast cancer cells, as evidenced by clinical studies, reviews, and trials. TP53 is a tumor suppressor and MDM2 facilitates proteasomal degradation of TP53. MDM2 and TP53 activity is tightly regulated. However, cancerous breast cells overexpress *MDM2* through five hypothesized mechanisms. Consequently, TP53 levels decrease with increased tumor cell proliferation. Three strategies have been identified for controlling *MDM2* upregulation in cells with wild-type or mutated TP53. MDM2 inhibitors (MDM2i) are administered in combination with existing chemotherapies to reduce their effects on healthy cells. Few clinical and preclinical studies have been conducted using MDM2i, which necessitates high-quality clinical trials to support their therapeutic potential in breast cancer therapy.

## Introduction

Breast cancer is a significant global concern, particularly for women. According to reports from the World Health Organization (WHO), breast cancer alone accounted for 2.3 million cases worldwide, causing 670,000 deaths in 2022 [1]. The mammary glands are bilaterally distributed and superficially positioned in the pectoralis major muscle [2]. They consist of lobules and a network of milk-producing epithelial structures. Lobules further aggregate to form lobes interspersed with adipose tissue. Cooper’s ligaments, which comprise fibrous connective tissue, provide structural support by anchoring the breasts to the underlying musculofascial layer [2]. In most cases, breast cancer occurs within the ductal epithelium and results in ductal carcinoma. However, some patients develop malignancy in the lobules called lobular carcinoma.

Breast cancer is estimated to affect one in eight women and accounts for one in every 39 deaths in females in the United States [3]. A recent study reported that breast cancer was the second most common cancer after lung cancer among new cancer in females in 2022 [4]. The predisposition towards developing breast cancer in women increases with age [5]. The incidence rate in women aged between 20 and 24 years is approximately 1.5 cases per 100,000 females, and surges to 421.3 cases per 100,000 in women aged 75–79 years. Only 5% of the reported cases involved women aged <40 years. The median age at breast cancer diagnosis is 61 years in women [5]. Therefore, breast cancer should be managed safely and effectively to reduce global comorbidities and mortality.

**Figure 1:**
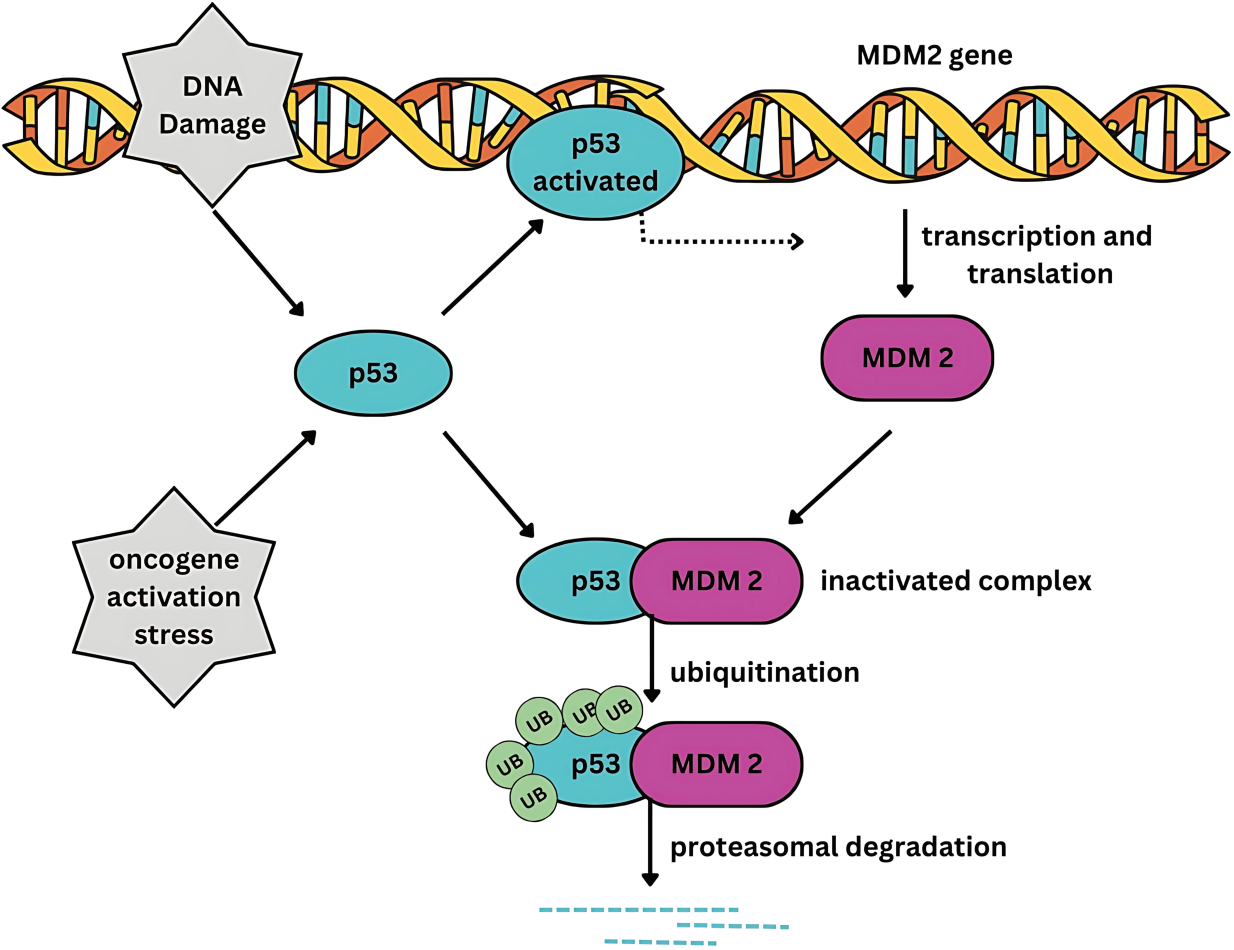
Feedback control of MDM2 and TP53. MDM2 tightly regulates the transcriptional activity of TP53. Conversely, TP53 positively regulates MDM2. MDM2 exerts its inhibitory influence on TP53 by binding to its transactivation domain, ubiquitinating TP53, thereby targeting it for proteasomal degradation. Under normal cellular homeostasis, a dynamic equilibrium exists between the transcriptional and MDM2-bound states of TP53 through a negative feedback mechanism.

Somatic mutations in tumor protein p53 (*TP53*), a tumor suppressor gene, are the most common genetic alterations in diverse malignancies. *TP53* mutations occur in approximately 50% of the known cancer types, including breast cancer [6,7]. The murine double-minute 2 proto-oncogene (MDM2) is a negative regulator of TP53, the primary tumor suppressor protein. Under normal cellular conditions, *TP53* expression is tightly regulated by MDM2. MDM2 mediates ubiquitination of TP53, tagging it for proteasomal degradation (Fig. 1). However, when cells experience stress, TP53 undergoes structural modifications, which increase its stability. Its phosphorylation stabilizes its structure, allowing its translocation into the nucleus where it acts as a transcription factor. TP53 facilitates the induction and downregulation of diverse genes involved in maintaining genomic integrity [8]. In addition, p53 regulates cell fate by inducing apoptosis, DNA repair, and cell cycle arrest. It regulates immunological processes such as inflammation, immune cell activation, and antigen presentation [9].

The TP53 pathway mediates several antiproliferative responses such as cell cycle arrest, apoptosis, and DNA repair. These criticalcellular processes are typically activated in response to various cellular stress signals [10]. *MDM2* is abnormally expressed in several malignant tumors. MDM2 activity is upregulated by gene amplification, which increases its transcription and translation. Because MDM2 facilitatesTP53 degradation, upregulation of MDM2 lowersTP53 activity and tumor suppression [11]. This relationship underscores the therapeutic potential of targeting the TP53-MDM2 interaction to restorating TP53 activity in breast cancers with wild-type or functionally intact TP53. This strategy also offers a unique advantage of activating the intrinsic tumor-suppressive machinery of cells [12]. This strategy differs from conventional approaches that focus on inhibition of oncogenic drivers. However, there is a gap in current theoretical knowledge and its translation into clinical treatments. Therefore, this review aims to investigate the efficacy of MDM2 inhibition in restoring TP53 activity in breast cancer cells, as evidenced by clinical studies, reviews, and trials. We also aimed to elucidate the regulatory function of MDM2 in breast cancer. Finally, this study provides a comprehensive overview of the role of MDM2 in breast cancer and the significance of therapeutic targeting of MDM2.

## Role of MDM2-TP53 in Breast Cancer Development and Progression

### Mutated TP53 in breast cancer cells

There is a strong association between TP53 activity in breast cancer cells and tumor development and progression. Breast cancer is heterogeneous and presents diverse subtypeswith distinct features, molecular profiles, and therapeutic responses. Across all breast cancersubtypes, one-third of patients have one or multiple *TP53* mutations [13], which are predominantly located within the DNA-binding domain. Mutations are often missense point mutations that disrupt the transcriptional activity of TP53 [14]. *TP53* mutations are significantly more common in patients with advanced breast cancer, high-grade tumors, and aggressive tumors such as the erb-b2 receptor tyrosine kinase 2 (ERBB2)^+^ subtype. *TP53* mutations are significantly more common in inflammatory (50%) than in noninflammatory (<30%) breast cancer cases [15].

A compelling association exists between *TP53* mutations in the DNA-binding domain and specific breast cancer subtypes [16]. These mutations are highly potent and significantly reduce patient survival rates. To understand the role of p53 in breast cancer, it is necessary to understand its exact impact. Breast cancer subtypes are classified as ERBB2^+^, luminal A, luminal B, and triple-negative [17]. Luminal-like breast cancer exhibits the lowest prevalence of p53 mutations, with 26% in luminal-A and 17% in luminal-B. ERBB2^+^ breast cancer has an intermediate mutation rate of 50% [18]. Concurrent TP53 mutation and ERBB2 expression correlated with significantly more adverse progression than subtypes. The highest prevalence rate (88%) of p53 mutations was observed in basal-like breast cancer [18]. In a recent study, mutated TP53 was found to potentially result in reduced survival time in patients with metastatic breast cancer, irrespective of the subtype [19]. Therefore, TP53 mutations can increase mortality and comorbidities. As TP53 controls *MDM2* expression via a feedback mechanism, this imbalance in TP53 and MDM2 activity results in malignant changes in healthy cells. *MDM2* is overexpressed in cancer cells in 7% of all malignancies [20].

### Wild-type TP53 and MDM2 activity in breast cancer cells

While *TP53* is often mutated in breast cancer cells, two-thirds of patients do not have *TP53* mutations. In patients with wild-type TP53, the lack of TP53 activity is due to the overexpression of *MDM2*, which suppresses the anti-tumor function of TP53 [14]. MDM2 treatment is specific to breast cancer patients with wild-type TP53, because of the modified binding of TP53 to MDM2. Under normal physiological conditions, mature breast epithelia preserve their genetic integrity with equilibrated MDM2 and TP53 activity. When DNA is damaged by exposure to radiation, mutagens, carcinogens, or post-translational modifications, MDM2 binds less effectively to TP53 [21]. MDM2 undergoes ubiquitination and subsequent proteasomal degradation, facilitating the accumulation of p53 and the activation of DNA damage repair pathways.

Upon activation under stress conditions, TP53 induces cell cycle arrest and triggers a cascade of DNA repair mechanisms when genomic repair is required [22]. The prominent feature of TP53 and MDM2 is their feedback loops. TP53 functions as a transcription factor that upregulates the expression of numerous target genesincluding *MDM2*. TP53 activation increases MDM2 levels, manifesting as an oscillatory feedback loop and enabling the restoration of basal TP53 levels after the resolution of cellular stress.

#### Cellular mechanism guiding MDM2 expression changes in breast cancer cells

**Figure 2:**
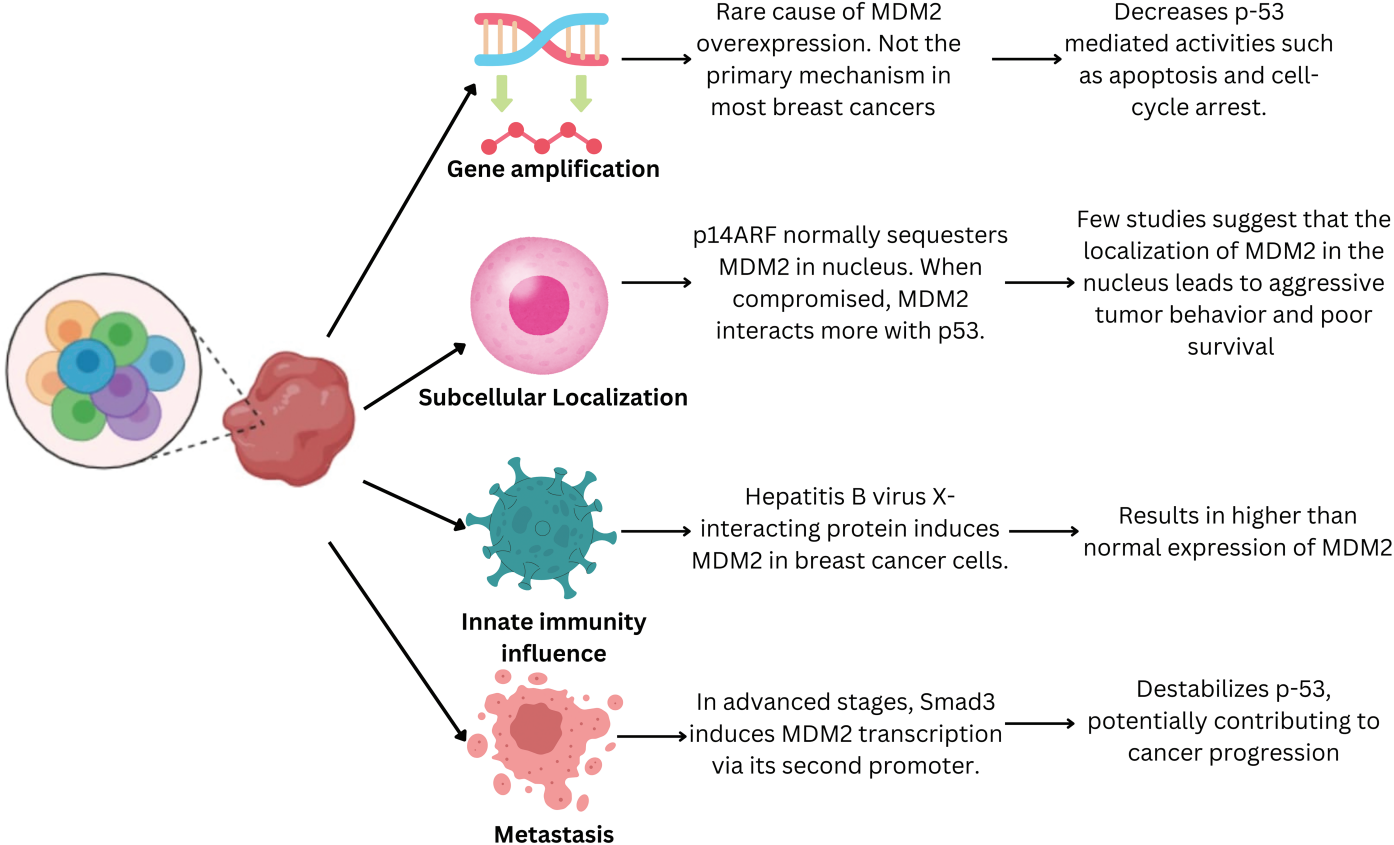
Pathway leading to increased MDM2 expressionin breast cancer cells. MDM2 is upregulated in 38% of patients with breast cancer via multiple mechanisms. Its overexpression may be a cellular mechanism to evade TP53-mediated growth suppression. These mechanisms lead to enhanced MDM2 activity, potentially promoting breast cancer development and progression.

*MDM2* is upregulated in 38% of patients with breast cancer. However, this increase cannot be explainedby gene amplification alone. Several mechanisms have been proposed to contribute to TP53 degradation in breast cancer cells [23] (Fig. 2). The least responsible pathway was found to be increased gene transcription. Studies have reported that *MDM2* overexpression is rarely caused by increased *MDM2* gene copy number. Breast cancer cells with estrogen and progesterone receptors showed *MDM2* overexpression in wild-type TP53. Therefore, increased *MDM2* expression cannot be attributed to coding alterations in most patients [24].

*MDM2* overexpression may also be attributed to a mechanism that evades TP53-mediatedgrowth suppression. Dysregulation of the subcellular localization of MDM2 is another critical factor that promotes enhanced MDM2 activity in human breast cancer cells [23]. Under normal physiological conditions, the cyclin-dependent kinase inhibitor 2A (CDKN2A/p14^ARF^) tumor suppressor protein sequesters MDM2 within the nucleus and restricts its interaction with TP53. However, it has been reported that p14^ARF^ function is compromisedin a significant proportion of human breast cancer cells owing to various genetic aberrations such as promoter hypermethylation, deletions, and loss of heterozygosity [25].

Another hypothesized mechanism for p53 inhibition by MDM2 is modification of the genetic regulators of MDM2, which leads to its overexpression. Studies suggest *MDM2* is overexpressed in breast cancer cells positive for estrogen receptor 1 (ESR1/ER-alpha). A specific polymorphism in *MDM2* (SNP309) markedly increases the risk of developing breast cancer [26]. The SNP309 polymorphism increases the binding affinity of the Sp1 transcription factor, leading to the overexpression of MDM2 [27]. A study of Caucasian women found that *MDM2* with a mutant C allele for the SNP285 polymorphism was less susceptible to overexpression and protected against breast cancer [28]. Therefore, the presence or absence of specificestrogen receptor genotypes may modulate susceptibility to developing breast cancer. However, more controlled high-quality evidence is required to confirm this hypothesis.

The next mechanism that modulates MDM2 activity is through the presence of certain viruses. Li et al. observed elevated MDM2 levels in patients with hepatitis B [29]. Hepatitis B virus X-interacting protein (HBXIP) is a potent inducer of *MDM2* in breast cancer cells. This induction appears to be mediated by direct binding of HBXIPto TP53 at the P2 site in the *MDM2* promoter, activating its transcription [29]. In patients with breast cancer in the advanced stages of metastasis, SMAD family member 3 (SMAD3) has been implicated in inducing *MDM2* transcription via its second promoter [30].

## Strategies Using MDM2 Inhibition as a Treatment Intervention for Breast Cancer Patients

### Therapeutic potential of MDM2 in modulating breast cancer

**Figure 3:**
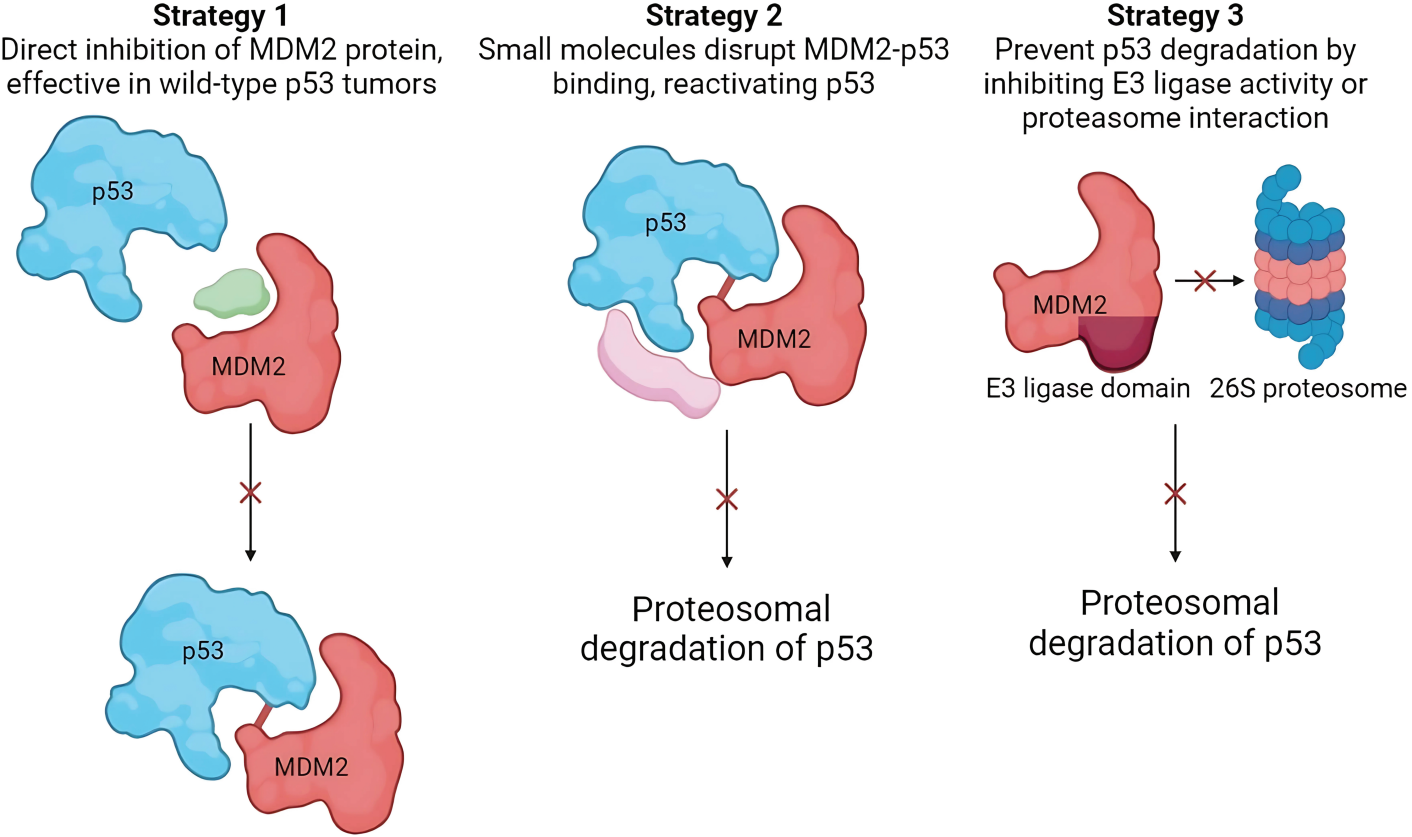
Therapeutic strategies targeting MDM2 in breast cancer. All three strategies converge on stabilizing and activating TP53, promoting its tumor-suppressive functions. These approaches offer promising avenues for breast cancer therapy (Image created with Biorender).

The oncogenic behavior of MDM2 is caused by its overexpression. The oncogenic properties of MDM2, owing to its overexpression, have been detected in approximately 38% of breast cancer patients [31]. Therefore, MDM2 is a compelling therapeutic target, particularly for tumors harboring wild-type TP53. Current therapeutic strategies against MDM2 primarily focus on three key approaches: (i) inhibiting the MDM2-TP53 interaction to preventTP53 inactivation, (ii) downregulating *MDM2* expression to reduce its inhibitory effect on TP53, and (iii) inhibiting MDM2 E3 ubiquitin ligase activity to disrupt its ability to target TP53 for proteasomal degradation. All three strategies aimed to stabilize TP53 and activate downstream tumor-suppressive pathways (Fig. 3).

The first strategy to inhibit MDM2 activity is blocking the MDM2-TP53 interaction, prevent TP53 inactivation. Baek et al. explained the rationale behind this approach. Their study observed a hydrophobic surface pocket at the sequences Phe19, Trp23, and Leu26 in the MDM2-p53 bound complex [32]. This pocket serves as a potential binding site for small molecules that disrupt the MDM2-TP53 interaction and consequently reactivate TP53’s tumor suppressor activity in cancer cells. More than 20 chemical classes have been shown to exhibit MDM2-TP53 inhibitory properties. Nutlins are the most frequently studied class of chemicals.

The second strategy involves direct disruption of MDM2 activity through its downregulation. This strategy is extremely advantageous for tumor cells with wild-type TP53 [14]. Implementing such a strategy would result in an outcome similar to that of the previous strategy. However, their mechanisms of action differ from each other. Instead of inhibiting the MDM2-TP53 complex, it directly acts on MDM2. Chemicals with affinity for MDM2 can bind to MDM2, leading to decreased activity or degradation. The compounds used in this strategy were JapA, Inulanolide A linearifolianoids.

The third strategy is to inhibit the proteasomal degradation activity of the MDM2 E3 ligase domain [14]. Therapeutic interventions can potentially target the E3 ubiquitin ligase activity of MDM2 or its interaction with the 26S proteasome complex to impede degradation of wild-type TP53. MDM2 is a well-established E3 ligase that concurrently promotes the degradation of various tumor suppressor proteins, including retinoblastoma protein (RB) and p53 [33]. Therefore, inhibition of E3 ligase activity results in anti-tumor activity potentially manifesting through mechanisms along with p53 activation, which are not well defined. All three strategies focused on downregulating MDM2 activity and promoting the tumor-suppressive activity of TP53. A prominent example of a compound that uses this strategy is sempervirine.

### Combination of MDM2 inhibition with chemotherapy

Several MDM2 inhibitors (MDM2i) restrict the activity of MDM2. MDM2i can be classified based on their categories as stapled peptides (ALRN-6294), cis-imidazoline (RG7112), pyrrolidine (idasanutlin), spirooxindole (milademetan), and piperidinones (navtemadlin) [34–38]. With an emerging understanding of the role of MDM2 overexpression in breast cancer progression, a novel finding is the ability of MDM2i to reverse chemotherapeutic resistance, in addition to its established role in facilitating cell cycle arrest and activating apoptotic pathways. Paclitaxel is a chemotherapeutic agent widely used in the standard treatment regimen for breast cancer. Its mechanism of action involves the promotion of microtubule polymerization, resulting in microtubule stabilization, which triggers mitotic arrest and leads to cancer cell death [39]. Similarly, eribulin is an FDA-approved microtubule-inhibiting chemical that has demonstrated safety and tolerability, with clinical studies indicating significant improvements in both all-cause mortality and progression-free survival in patients with advanced-stage breast cancer who have experienced disease progression even after undergoing other chemotherapies [40]. Similar instances have been noted for doxorubicin, an effective first-line therapy in breast cancer patients, belonging to the class anthracycline. Doxorubicin resistance increases the risk of cancer recurrence and metastasis. As MDM2 is a well-identified facilitator of chemoresistance, it is necessary to search for novel adjuvants that can potentially reverse this resistance and increase the sensitivity of breast cancer cells towards doxorubicin. An *in vitro* study by Fan et al. reported that cell cultures co-administered with doxorubicin and MDM2i exhibited reduced expression levels of P-glycoprotein, multidrug resistance-associated protein, cdc2, and Bcl-2, in addition to elevated expression of Cyclin B1 and Bax [41]. These findings provide evidence of the arrest of mitosis in the multi-drug resistant breast cancer cell lines. These molecular alterations appear to result from the activation of various signaling cascades, as evidenced by small interfering RNA (siRNA)-mediated silencing experiments and immunohistochemical analysis of breast cancer tissue samples. Additionally, an inverse relationship was identified in patients with breast cancer, where high expression levels of MDM2 and murine double minute X (MDMX) corresponded with reduced TGF-Beta Activated Kinase 1 (MAP3K7) Binding Protein 1 (TAB1) expression.

The potential action of MDM2 against breast cancer types that show resistance to different therapeutic agents has recently been studied. Pairawan et al. studied the effects of MDM2i on estrogen receptor (ER)-positive breast cancer cells *in vitro* [42]. Their study was based on the rationale that MDM2 overexpression facilitates taxane resistance and improves the invasive capacity and mobility of breast cancer cells. The study findings reported that MDM2i conjugated with chemotherapeutic agents paclitaxel and eribulin successfully enhanced the anti-tumorigenic response of cancer cells. Additionally, MDM2i, either as monotherapy or in combination with paclitaxel, reactivates p53 function, thereby inhibiting cell cycle progression primarily through p21-mediated mechanisms [42].

An unexpected finding of this study was the off-target effect of MDM2i on a protein called mammalian target of rapamycin (mTOR). When the MDM2i was combined with paclitaxel, p-mTOR was downregulated, providing a possible rationale for the observed inhibition of breast cancer cell growth. The mTOR pathway is a well-established signaling cascade involved in the proliferation and metastasis of breast cancer cells. Recent studies have identified a significant role for the mTOR pathway in the development of resistance to endocrine therapies, particularly in advanced stages of breast cancer [43]. Inhibition of this pathway has been shown to reverse resistance and improve the sensitization of estrogen-positive breast cancers. Clinically, the mTOR inhibitor everolimus has been demonstrated to improve progression-free survival when combined with exemestane, compared to exemestane monotherapy, in postmenopausal females with advanced-stage breast cancer [44]. This confirms that mTOR is a critical therapeutic target in breast cancer.

Fan et al. used three triple-negative breast cancer cell lines to assess the antitumor activity of MDM2i and doxorubicin [45]. A novel dual-target MDM2/MDMX inhibitor employing *Escherichia coli*-derived proteins improved the transduction of chemotherapeutic agents across breast cancer plasma membranes. The combination of an MDM2/MDMX inhibitor and doxorubicin potentially arrests the cell cycle of chemotherapeutic-resistant cells at the G2/M phase by inducing p21 expression. Furthermore, treatment with the MDM2i alone significantly arrested the cell cycle in the G2/M phase in chemotherapeutic-resistant cells, a response that was not observed with doxorubicin treatment alone. This suggests that cell cycle effects are primarily driven by MDM2i [41].

### Combination of MDM2 inhibition with immunotherapy

The progression and prognosis of breast cancer, like any other tumor, are highly regulated by the immune response and the tumor microenvironment. The tumor microenvironment comprises a complex network of immune cells, extracellular matrix components, stromal cells, vasculature, and secreted compounds, all of which collectively influence tumor progression [46]. The tumor microenvironment can either suppress or promote tumor growth depending on the relative abundance of immunosuppressive and immune-supportive factors [47]. These factors vary significantly according to organ type, tumor type, cancer stage, and demographics specific to the individuals [48]. In the majority of advanced solid tumors, the tumor microenvironment is predominantly composed of immunosuppressive elements. The inactivation or loss of p53 plays a pivotal role in shifting the tumor microenvironment toward a pro-tumorigenic inflammatory state, whereas the reactivation of p53 can restore immune activity and counteract suppression [49]. Therefore, as MDM2 is a key negative regulator of p53, it directly and indirectly modulates the tumor microenvironment, primarily by inhibiting p53 function [50].

Apart from the role of MDM2 in the microenvironment of breast cancer cells, it also affects other factors such as immune checkpoint inhibitors. Breast cancer cells can potentially evade immune checkpoints by secreting compounds with a high binding affinity towards T-cells, evading T cell-mediated immune responses, and degradation [51]. Therefore, the secretion of certain binding agents with T cell receptors and the consequent inactivation is a major determinant of cancer endurance. Consequently, immune checkpoint inhibitors have been explored as promising therapeutic options to counteract tumor survival. Nonetheless, resistance to immune checkpoint inhibitors develops in the majority of patients receiving these treatments, and hyperprogressive disease represents a significant barrier to a clinical response [52]. In this context, overexpression of MDM2 contributes to immune evasion through various mechanisms, with studies demonstrating a positive correlation between MDM2 overexpression and hyperprogressive disease in cancer cells [53].

Guo et al. revealed that the activation of TP53 as a result of Nutlin (an MDM2i) results in damage-associated molecular pattern production, causing immunogenic apoptosis in cancer cells [54]. Furthermore, studies have demonstrated that the blocking of MDM2 in cancer cells is associated with an immune response against cancer mediated through the Fas/FasL pathway [55]. Therefore, it is hypothesized that MDM2 inhibition can help overcome the immunosuppressive activity of breast cancer cells. However, further studies are required to confirm this hypothesis, which may revolutionize the response to breast cancer treatment.

#### Differential performance of MDM2i in different breast cancer types

MDM2i successfully overcomes chemotherapeutic resistance in ER-positive breast cancer (1). A recent *in vitro* study by Portman et al. assessed the role of MDM2i in conjugation with endocrine therapy, which is the mainstay treatment for ER-positive breast cancer cells [56]. Endocrine therapy effectively complements MDM2 inhibition, leading to synergistic effects, primarily through the modulation of cell cycle regulatory pathways. Combination treatment regulates transcriptional programs associated with cell cycle control, achieving synergy by intensifying the suppression of cell cycle progression. This therapeutic approach induces senescence in cell lines that display resistance to either fulvestrant or palbociclib, thereby significantly inhibiting tumor proliferation *in vitro*. Therefore, further human studies are warranted.

Aggressive cancers, such as triple-negative breast cancer (TNBC), often demonstrate resistance to drugs, making it a prominent cause of morbidity and mortality among other breast cancers [57]. It also has high recurrence and metastasis rates [58]. Targeting MDM2 as a strategy for controlling TNBC progression has shown promising results. Adams et al. reported increased expression of p53 following the use of an MDM2-(PROteolysisTArgeting Chimera) PROTAC in mouse models, which induced apoptosis of TNBC cells while sparing healthy cells [59]. This study identified the role of TAp73 in facilitating MDM2-mediated apoptosis in TNBC cells.

#### Uses of MDM2 as a prognostic marker in breast cancer

The role of MDM2 as a prognostic marker has been studied, but its clinical translation is lacking. A recent *in silico* investigation by Zheng et al. in an attempt to correlate MDM2 overexpression with patient outcomes observed that MDM2 was highly predictive of a worse prognosis [60]. Furthermore, their study reported a correlation between MDM2 overexpression and tumor mutational burden, indicating a higher risk of underlying mutations in MDM2 overexpressed cells. MDM2 overexpression also translates to microsatellite instability and drug sensitivity in some breast carcinoma subtypes. Breast cancer cells overexpressing MDM2 demonstrate a significantly increased capacity for immune cell infiltration [60]. However, the impact of MDM2i on immunotherapy outcomes may vary depending on the infiltrating immune cell types, as cytotoxic cells could enhance therapeutic efficacy, whereas immunosuppressive cells may hinder it.

MDM2 mRNA was found to be significantly overexpressed in breast cancer cells compared with non-cancerous breast tissues. This finding was confirmed by immunohistochemistry, which showed MDM2 overexpression in 24 out of 33 tumors analyzed, despite the absence of gene amplification [61]. Several MDM2 mRNA isoforms that are not typically present in normal breast tissue have been identified and their expression is associated with poor clinical outcomes [62]. Further correlation between MDM2 overexpression and estrogen receptor α status revealed that while MDM2 overexpression was specific to estrogen receptor α-positive tumors, the same was not true for MDM2 mRNA splice variants. Notably, elevated MDM2 levels are associated with a more favorable prognosis in estrogen receptor-positive tumors. Studies using tissue culture models have demonstrated that p53-independent activation of MDM2 expression in breast carcinoma cell lines is mediated by the estrogen receptor α status, occurring mainly in estrogen receptor α-positive cells. This suggests the involvement of p53-independent mechanisms in regulating the p53 pathway through modulation of MDM2 expression. An *in vitro* study found increased MDM2 expression in estrogen receptor α-positive cells, inducing the P2 promoter, which is independent of p53 [63]. In summary, the improved prognosis and survival of patients with MDM2 overexpression appears to be limited to estrogen receptor α-positive breast cancer patients, likely driven by p53-independent activation of MDM2.

Fulvestrant, which is a commonly employed selective estrogen receptor degrader, has been documented to reduce estrogen-mediated upregulation of MDM2 and to accelerate the turnover rate of MDM2 protein, with no effect on *MDM2* expression or p53 function. Notably, MDM2 reduction occurs irrespective of p53 mutation status [14]. Another p53 independent pathways involves early region 2 binding factor (E2F), a transcription factor essential for regulating the cell cycle. The p53 and E2F transcription factors exhibit both antagonistic and cooperative interactions. This interaction is potentially modulated by MDM2 as it regulates E2F negatively and positively through p53 independent pathways. Klein et al. observed cell cycle arrest in p53 mutant cancer cells due to a decrease in the expression of E2F proteins due to loss of MDM2 activity [64].

A study conducted by Park et al. aimed to understand the prognostic value of MDM2 based on its subcellular localization [65]. Tissue microarrays of 865 patients were assessed by immunohistochemistry for co-expression of p53 and MDM2. The results of their Multivariate analysis showed that the simultaneous expression of MDM2 within the nucleus and cytoplasm is a statistically significant prognostic indicator in breast carcinoma [65]. In contrast, cytoplasm-positive cells showed better prognosis, underscoring the importance of MDM2 localization in cancer progression. Similarly, other studies have suggested that MDM2 exerts distinct regulatory effects on p53 activity depending on its concentration in the nucleus [66]. Specifically, low nuclear levels of MDM2 facilitate p53 sumoylation, which may modulate p53 activity, whereas elevated nuclear Mdm2 levels promote p53 degradation [66]. Given these findings, assessing both nuclear and cytoplasmic MDM2 expression may provide a more accurate prognostic indicator in breast cancer patients than solely evaluating the p53 status. This suggests that combined analysis of MDM2 localization and expression patterns could offer valuable insights into disease progression and patient outcomes.

## Clinical and Preclinical Studies on MDM2 in Breast Cancer Therapy

### Preclinical studies on MDM2 in breast cancer

Alaseem et al. carried out an *in vitro* investigation of MDM2i idasanutlin in the MCF-7 breast cancer cell line [67] andcompared it to the well-known chemotherapeutic agent axitinib. They demonstrated that axitinib inhibited MCF-7 cell proliferation at ahalf-maximal inhibitory concentration (IC_50_) of approximately 30 µmol/L. Concurrently, idasanutlinalso showed cytostatic effects in MCF-7 cells, exhibiting a significantly lower IC_50_ of approximately 10 µmol/L, inducing cell death in cancer cell lines by facilitating mitotic arrest and subsequent cell death. They also reported improved efficacy of combined treatment with idasanutlin and axitinib compared with monotherapy in inducing cell death. Therefore, theyconcluded that dual therapy is a better treatment intervention, particularly for active compounds that target different moieties. Combination therapy can improve the safety of both drugs without decreasing their therapeutic potential [67].

Adams et al. developed a novel therapeutic agent, PROTAC [59]. They tested their innovation in a mouse model of triple-negative breast cancer. They found an unusual phenomenon in which triple-negative breast cancer cells required MDM2 for survival in the absence of wild-type TP53 [59]. Therefore, MDM2 inhibition translates into a direct apoptotic effect in these cells, whereas healthy cells with non-mutated TP53 are unaffected by PROTAC. This study provides evidence for this hypothesis, paving the way for another dimension of the MDM2-TP53 interaction in breast cancer cells.

PROTACs were initially conceptualized over two decades ago, originating in 1999 and advancing with the synthesis of the first PROTAC targeting methionine aminopeptidase-2 (MetAP-2) in 2001. However, there has only recently been an accelerated surge in the development of these molecules, making them promising candidates for effective cancer therapies [68]. The mechanism of action of PROTACs transcends their bifunctional activity, which facilitates the targeted post-translational degradation of specific proteins (POIs) by leveraging the cell’s endogenous ubiquitin-proteasome system (UPS) [69]. The primary function of PROTACs is to spatially position a POI near an E3 ligase enzyme that tags the POI with ubiquitin molecules. This process labels proteins for recognition and subsequent degradation by the 26S proteasome. For effective degradation, the POI must undergo polyubiquitination, where multiple ubiquitin molecules are sequentially attached to generate a ubiquintin chain [70]. Initially, the first PROTAC, developed based on MDM2 E3 ligase, demonstrated the advantage of achieving substantial molecular size reduction. However, researchers have demonstrated the potential of utilizing MDM2 in cancer treatment. This was identified as a recruiting ligase offering additional therapeutic benefits in oncology due of MDM2’s role in regulating the tumor suppressor protein p53, a key element in the pathogenesis of numerous cancers [71,72].

Singh et al. conducted an *in silico* search for compounds that could affect multiple targets in triple-negative breast cancer cells [73]. Topoisomerase II and MDM2-TP53 complex were used in their study. Bioactive compounds that efficiently bind to this complex would subsequently block TP53 degradation by increasing the concentration of wild-type TP53 in cells. The compound that showed high binding affinity for this complex was identified as resveratrol based on molecular docking. This study can be used to understand the therapeutic potential of MDM2i and to guide further studies to determine its utility in managing breast cancer. As triple-negative breast canceris a challenge for clinicians and patientsglobally, with limited therapeutic options, and this study proposes resveratrol as an effective alternative to current treatment options.

A recent study proposed a novel approach to target mutated GATA-binding protein 3 (GATA3) by modulating MDM2 activity using small molecules [74]. The authors used an *in silico* approach to identify lethal GATA3 mutants in different breast cancer lines based on RNA interference data from other studies and identified 13. The rationale behind identifying these variants was to predict the outcomes of MDM2i treatment in patients with breast cancer. A significant finding was the differential response of patients with wild-type and mutated GATA3. Cell lines expressing wild-type GATA3 showed a rapid apoptotic response after idasanutlin treatment. GATA3 was rescued by molecular methods to confirm this hypothesis. Proliferation was significantly higher in these cells than in wild-type cells, indicating the sensitivity of MDM2i cells to GATA3. Therefore, a patient could be genetically analyzedbefore starting MDM2i in conjugation with chemotherapyto predict their clinical outcomes and assess whether they would benefit from this strategy. Chemotherapy involves various factors, and important drug interactions must be considered.

ALRN-6924 is another class of MDM2/MDMX inhibitor that enhances the antitumor activity of chemotherapy. Recently, various clinical trials have been conducted to investigate this novel approach. Findings from earlier breast cancer models indicate that this therapeutic agent ALRN-6924 significantly potentiates the antitumor effects of paclitaxel and eribulin in both *in vitro* and *in vivo* models [42]. Additionally, the study observed that ALRN-6924, whether administered alone or in combination with paclitaxel, reinstates p53 functionality and impedes cell cycle progression, primarily via the upregulation of p21 activity. The elevation in phosphorylated histone H3 (p-HH3) levels following paclitaxel monotherapy aligns with prior findings, suggesting a cellular response to cytotoxic stress and an ensuing apoptotic process [42].

### Clinical trials on MDM2 in patients with breast cancer

MDM2i has not been widely investigated as a potent anti-tumor drugin the existing clinical landscape. Few studies have investigated their activities in other cancer types. However, these studies have pioneered the relevance of MDM2i in patients with breast cancer by elucidating its efficacy, toxicity profiles, dosages, and other relevant parameters. One clinical trial that began in 2021 (NCT03725436) focused on MDM2i in patients with advanced-stage breast cancer [75], including those with metastasis to different organs, rendering surgical removal ineffective. The patients were administeredan MDM2i, ALRN-6924, in combination with paclitaxel. It assesses the maximum tolerated dose and other secondary factors such as pharmacokinetics, overall survival, progression-free survival, dose-related responses, genetic changes, and cytology [75]. The project is intended to be completed by May 2025. To the best of our knowledge, no study has been published on this clinical trial.

Another clinical trial (NCT02264613) also investigated the response of tumor cells to ALRN-6924 [76]. However, the study population included patients with diverse solid tumors. It included three patients with breast cancer, of whom one showed stable disease with an approximately 20% increase in tumor size, one showed a reduction in tumor size but elevated MDM2 levels, and one showed a progressive tumor increase of >20%. The efficacy of MDM2i in decreasing tumor sizewas weaker in patients with breast cancer than in those with other cancers. However, the small number of patients with breast cancer indicates that these findings must be interpreted cautiously. One significant finding was the favorable tolerability profile of ALRN-6924, with a decreased negative impact on healthy cells, whereas apoptosis was not observed in non-cancerous cells at the highest dose administered. This finding suggests that ALRN-6924 can be used at low doses to prevent chemotherapy toxicity. This phenomenon is referred to as cyclotherapy and has been reported previously [77,78].

Another clinical trial (NCT05622058) also examined the effect of ALRN-6924 in patients with breast cancer [79]. In this study, researchers aimed to assess the protective effects of MDM2i in healthy cells by evaluating the safety and tolerability of the treatment. However, it was terminated due to the development of grade IV neutropenia and failure to meet the primary outcome. Therefore, no results have been published to date. Nonetheless, the rationale was to evaluate the protective effect of MDM2i in breast cancer patients undergoing chemotherapy. Chemotherapeutic agents exert cytotoxic effects predominantly on proliferating cells, targeting both neoplastic and normal tissues that undergo cell division. ALRN-6924 is hypothesized to selectively inhibit cell division within normal tissues, while sparing tumor cells withTP53 mutations. This temporary arrest of cell cycle protects normal cells from the activity of chemotherapeutic pathways that target cells undergoing cell division. It further preserves the integrity of normal tissues and mitigates adverse effects commonly associated with chemotherapy [79]. Therefore, it increases the safety of chemotherapy and reduces its side effects.

An additional clinical trial (NCT03566485) investigated the activity of the MDM2 iidasanutlin, an MDM2 antagonist in breast cancer cells [80]. This study specifically included patients with ER-positive breast cancer. The study participants also had a mutated *TP53* gene. Seven patients were recruited, but none completed the study, with four showing disease progression, one showingclinical progression, and two developing acute toxicities. All the included participants reported adverse events that led to trial termination. This study did not provide definitive results because of the small study population [80].

## Conclusions

Despite advances in the understanding of MDM2i, its clinical application in patients with breast cancer faces significant challenges. MDM2i can effectively disrupt the cell cycle in p53-proficient cells but often fails to induce cell death, making combination therapies essential to achieve antitumor effects. Cyclotherapy, for example, could utilize MDM2i’s protective effects on healthy cells under cytotoxic treatment. Intriguingly, MDM2i may also exhibit p53-independent actions, with some studies showing molecular effects in p53-deficient cells, suggesting broader potential applications than previously understood. Overall, while MDM2i holds promise as a selective and protective therapeutic strategy, further high-quality research and large-scale human trials are needed to clarify its mechanisms, optimize combination regimens, and address safety concerns, including toxicity. These studies are essential for translating MDM2i from preclinical insights into viable clinical options for breast cancer treatment.

## Data Availability

The manuscript includes all the necessary data; related data may be provided upon request from the corresponding author.
